# Real-world treatment outcomes from a retrospective cohort of patients with acute myeloid leukemia from an urban safety net hospital

**DOI:** 10.1177/10781552231225398

**Published:** 2024-02-06

**Authors:** Joseph P Marshalek, Raisa Epistola, Sarah Tomassetti

**Affiliations:** Division of Hematology/Oncology, Department of Internal Medicine, 21640 Harbor-UCLA Medical Center, Torrance, CA, USA

**Keywords:** Acute myeloid leukemia, retrospective, real-world, ethnicity, disparities

## Abstract

**Introduction:**

While continual advancements in acute myeloid leukemia have augmented response rates and survival, outcomes in clinical trials may not correlate with real-world practice as trials may underrepresent individuals with comorbidities, decreased performance status, and older age. Additionally, clinical trials may underrepresent certain ethnicities, and disparities based on ethnicity, socioeconomic status, and insurance have been demonstrated in acute myeloid leukemia.

**Methods:**

We performed a retrospective chart review of adult patients with acute myeloid leukemia who were treated at Harbor-UCLA from 2014 to 2022 to examine patient characteristics, management patterns, and outcomes in a safety net hospital setting.

**Results:**

The median age was 56 years old (range 18–84). In regards to risk stratification, 22%, 33%, and 41% had favorable, intermediate, and adverse risk acute myeloid leukemia, respectively. The most common induction regimens included 7 + 3 (55%), azacitidine (10%), azacitidine + venetoclax (7%), and 7 + 3 + midostaurin (7%). The complete remission rate was 51%. Among patients who received intensive induction chemotherapy, 15% underwent re-induction with a second cycle, 51% received consolidation therapy, and 5% received maintenance therapy with a targeted agent. Overall, 12% of patients received allogeneic stem cell transplant. Median overall survival was 12.2 months, and 5-year overall survival was 18%.

**Conclusions:**

Suboptimal response rates and survival in this population may be related to low rates of re-induction and allogeneic transplant in addition to high rates of adverse cytogenetics, secondary acute myeloid leukemia, and supportive care only. Efforts to increase access to clinical trials, novel therapies, and transplants for diverse and underinsured populations are essential.

## Introduction

Over the last 40 years, five-year overall survival (OS) for acute myeloid leukemia (AML) patients has increased from 5%–10% to 30%^1^ due to advancements in bone marrow transplant and supportive care as well as the recent influx of targeted agents and novel therapies. Hypomethylating agents (HMAs) and venetoclax have vastly improved survival in patients who are older or ineligible for intensive induction chemotherapy.^2–5^ Targeted agents such as fms-related receptor tyrosine kinase 3 (FLT3) inhibitors,^6,7^ IDH1/2 inhibitors,^8^ and gemtuzumab ozogamicin^9–12^ have also enhanced outcomes for AML patients.

Despite continued evolution in AML treatments and improvements in survival, there remain disparities in access to treatment and outcomes based on ethnicity, race, socioeconomic status, and insurance. Multiple studies have shown that black and Hispanic patients with AML have inferior survival compared to white patients.^13–17^ Furthermore, black and Hispanic patients are less likely to participate in clinical trials^18^ and less likely to receive allogeneic stem cell transplants (SCTs).^19^ Lower socioeconomic and insurance status have also been associated with worse outcomes in AML.^14,16^ Efforts to explore these disparities are essential.

Treatment and outcomes in the real-world setting often fail to correlate with what is observed in clinical trials, as trials tend to exclude patients with organ dysfunction, older age, and poor performance status. Minimal research exists regarding AML management in the safety net hospital setting, and there is a particular paucity of research in Hispanic and Asian populations. The term “safety net hospital” refers to a medical center with a stated mission to provide medical care to patients regardless of insurance status or ability to pay. Herein, we retrospectively analyzed a cohort of 58 AML patients treated at a Los Angeles County safety net hospital including patient characteristics, management patterns, and outcomes.

## Methods

### Study design

Using the Cerner electronic health record, we performed a retrospective chart review of patients with AML age 18 years or older who were treated at Harbor-UCLA Medical Center from 2014 to 2022. Institutional Review Board (IRB) approval was obtained, and informed consent was waived due to the retrospective nature of the study.

### Patient inclusion criteria

The diagnosis of AML was based on the presence of 20% or greater myeloid blasts in the bone marrow or peripheral blood or recurrent genetic abnormalities consistent with a diagnosis of AML. Patients with therapy-related AML and AML with prior myelodysplastic syndrome (MDS) or myeloproliferative neoplasm (MPN) were included. Patients with acute promyelocytic leukemia were excluded, as were patients seen at Harbor-UCLA for a brief time without receiving definitive treatment or supportive care.

### Bone marrow biopsy analysis

Immunohistochemistry and flow cytometry were performed on-site in the Harbor-UCLA Hematopathology Lab (Torrance, CA). Cytogenetic and molecular analysis was performed by outside accredited laboratories. Chromosomal analysis was done by Quest Diagnostics Nichols Institute (San Juan Capistrano, CA). Molecular genetics was done by Quest Diagnostics Nichols Institute or Tempus Labs (Chicago, IL).

### Assessment of clinical response

Complete remission (CR) was defined by bone marrow blasts <5%, absence of circulating blasts, and absence of extramedullary disease. Patients with CR with incomplete hematologic recovery (CRi) met all CR criteria except with residual neutropenia (absolute neutrophil count <1 K/µL) or thrombocytopenia (<100 K/µL). Partial remission (PR) was defined by a decrease in bone marrow blast percentage to 5%–25% and by at least 50%. Patients with primary refractory disease failed to achieve CR, CRi, or PR after induction treatment. Relapse was defined as bone marrow blasts >5%, reappearance of peripheral blasts, or development of extramedullary disease after initial remission. CR rate (CRR) reported in this study refers to patients with CR or CRi.

### Risk stratification

Patients were classified as favorable, intermediate, or adverse risk according to European LeukemiaNet (ELN) 2022 risk stratification.^20^

## Results

### Patient demographics

The cohort of 58 patients consisted of 31 males (53%) and 27 females (47%) with a median age of 56 years old (range 18–84 years old). In regards to ethnicity/race, the study group was composed of 40% Hispanic, 26% Asian, 21% black/African-American, and 14% white/European patients (Table 1).

**Table 1.** Patient demographics, cytogenetics, molecular genetics, and outcomes stratified by ethnicity/race.

	All patients (*n* = 58)	Hispanic (*n* = 23)	Asian (*n* = 15)	Black/African-American (*n* = 12)	White/European (*n* = 8)
Median age (range) (years)	56 (18–84)	48 (18–77)	58 (21–84)	59 (24–83)	58 (29–79)
Sex, male	53%	61%	53%	50%	38%
ELN risk group					
Favorable	22%	22%	13%	25%	38%
Intermediate	33%	35%	40%	25%	25%
Adverse	41%	39%	47%	42%	38%
Unknown	3%	4%	0%	8%	0%
Mutations					
FLT3	26%	22%	20%	33%	38%
IDH1/2	12%	22%	7%	0%	13%
NPM1	24%	26%	13%	17%	50%
TP53	7%	0%	27%	0%	0%
AML type					
De novo	66%	74%	60%	75%	38%
Therapy-related	10%	9%	13%	8%	13%
Prior MDS/MPN	24%	17%	27%	17%	50%
Received treatment	88%	96%	87%	83%	75%
CRR (CR + CRi) to induction	51%	59%	38%	30%	83%
Relapse after initial CR/CRi	46%	46%	40%	33%	60%
Transplant					
Referred	36%	35%	40%	33%	38%
Received	12%	17%	13%	8%	0%
Median OS (months)	12.2	14.0	12.5	12.1	8.2
5-year OS	18%	31%	13%	0%	20%
Care out-of-network	47%	30%	53%	75%	38%
Lost to follow-up	14%	22%	13%	8%	0%

AML: acute myeloid leukemia; OS: overall survival; HMA: hypomethylating agent; FLT3: fms-related receptor tyrosine kinase 3; IDH: isocitrate dehydrogenase; MDS: myelodysplastic syndrome; MPN: myeloproliferative neoplasm; CR: complete remission; CRi: complete remission with incomplete hematologic recovery; PR: partial remission; CRR: complete remission rate; ELN: European LeukemiaNet.

### Clinical characteristics

A total of 20 patients (34%) had secondary AML (six therapy-related, 14 prior MDS/MPN), and 38 patients (66%) had de novo AML. The highest rate of secondary AML was observed in white/European patients (63%). In regards to ELN risk stratification, 22%, 33%, and 41% had favorable, intermediate, and adverse risk AML, respectively. A similar distribution of ELN risk was noted between ethnicities (Table 1).

**Figure 1.**
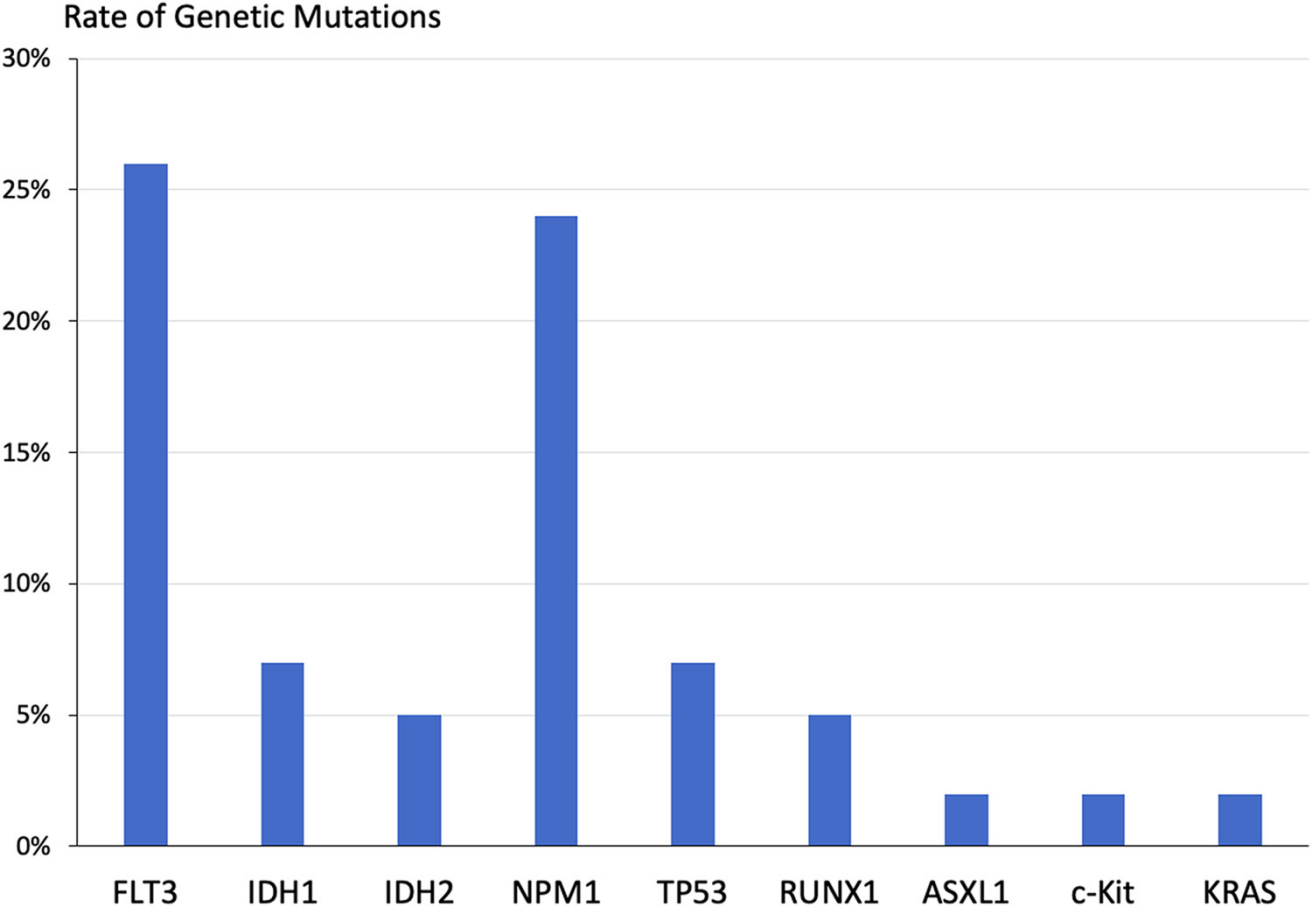
Genetic mutations were observed across all participants (*n* = 58).

The prevalence of FLT3 mutation was 26%, ranging from 20–38% across ethnicities. Seven patients (12%) had IDH1/2 mutations with a high rate observed in Hispanic patients (22%) and a low rate in black/African-American patients (0%). NPM1 mutations were relatively common (24%). Of note, all 4 patients with TP53 mutations were of Asian ethnicity. Figure 1 depicts the prevalence of all identified molecular alterations.

### Management

Prior to induction therapy, 36% and 3% of patients received hydroxyurea or leukapheresis, respectively, for cytoreduction. Of the 58 patients in the cohort, 51 patients (88%) received treatment, and seven patients (12%) received supportive care only. The median time from diagnosis to treatment initiation was 6 days, and the median inpatient days for induction was 27 days. The most common induction therapy was 7 + 3 (55%), which consisted of 7 days of cytarabine plus 3 days of anthracycline (the institutional standard was idarubicin). Other first-line regimens included 7 + 3 + midostaurin (7%), 7 + 3 + ivosidenib (2%), azacitidine (10%), azacitidine + venetoclax (7%), azacitidine + midostaurin (2%), AAML 1031 pediatric protocol (3%), and cytarabine (2%). Among 39 patients who received intensive induction chemotherapy, six patients (15%) underwent re-induction with a second cycle.

A total of 20 patients underwent consolidation with high-dose cytarabine (16 HIDAC and four HIDAC + midostaurin), and two patients received maintenance therapy with a targeted agent (one midostaurin and one ivosidenib). The rate of transplant referral was 36%, and 12% of patients received an allogeneic SCT.

Salvage therapies used in the second-line setting and beyond can be found in Table 2. The most common therapies utilized in the relapsed or refractory setting were HMA monotherapy (n = 6, 24%) and fludarabine + cytarabine + granulocyte-colony stimulating factor + idarubicin (FLAG-IDA, n = 5, 20%). Additionally, HMAs were used in combination with a FLT3 inhibitor (16%), venetoclax (12%), and venetoclax + FLT3 inhibitor (4%).

**Table 2.** Management patterns and response by number of patients.

First-line treatment	7 + 3 (*n* = 32, 58%)
	7 + 3 + midostaurin (*n* = 4, 7%)
	7 + 3 + ivosidenib (*n* = 1, 2%)
	Azacitidine (*n* = 6, 10%)
	Azacitidine + venetoclax (*n* = 4, 7%)
	Azacitidine + midostaurin (*n* = 1, 2%)
	AAML 1031 (pediatric protocol) (*n* = 2, 3%)
	Cytarabine (*n* = 1, 2%)
	Supportive care only (*n* = 7, 12%)
Response to first-line treatment	CR = 22 (43%)
	CRi = 4 (8%)
	PR = 6 (12%)
	Primary refractory disease = 17 (33%)
	Unknown = 2 (4%)
Consolidation therapy	HIDAC (*n* = 16, 28%)
	HIDAC + midostaurin (*n* = 4, 7%)
Maintenance therapy	Midostaurin (*n* = 1, 2%)
	Ivosidenib (*n* = 1, 2%)
Second-line therapy and beyond	HMA (*n* = 6, 24%)
	FLAG-IDA (*n* = 5, 20%)
	HMA + FLT3 inhibitor (*n* = 4, 16%)
	HMA + venetoclax (*n* = 3, 12%)
	HMA + venetoclax + gilteritinib (*n* = 1, 4%)
	Gilteritinib (*n* = 1, 4%)
	Enasidenib (*n* = 1, 4%)
	Cytarabine (*n* = 1, 4%)
	Cytarabine + clofarabine (*n *= 1, 4%)
	Etoposide + mitoxantrone (*n* = 1, 4%)
	7 + 3 + midostaurin (*n* = 1, 4%)

HMA: hypomethylating agent; FLT3: fms-related receptor tyrosine kinase 3; CR: complete remission; CRi: complete remission with incomplete hematologic recovery; PR: partial remission; HIDAC: high-dose cytarabine; FLAG-IDA: fludarabine + cytarabine + granulocyte-colony stimulating factor + idarubicin.

### Outcomes

Among 51 patients who received treatment, the CRR (CR + CRi) was 51% with 22 patients achieving CR (43%) and four patients with CRi (8%). An additional six patients (12%) had a PR. Asian patients (CRR = 38%) and black patients (CRR = 30%) had the lowest response rates to induction therapy. Among patients who achieved an initial CR or CRi, the rate of relapse was 46%.

The median OS was 12.2 months, ranging from eight to 14 months across ethnicities. Five-year OS was 18%. Hispanic patients had the highest 5-year OS (31%), and black patients had the lowest (0%). Overall, 27 patients (47%) received care out-of-network (as defined by outside the Los Angeles County Department of Health Services system) at some point throughout the course of their disease, and eight patients (14%) were lost to follow-up (Table 1).

### Bone marrow biopsy data

The median bone marrow blast percentage at diagnosis was 58% (Table 3). At day 14, most patients who underwent biopsy had a hypocellular marrow (89%). In contrast, many more patients demonstrated bone marrow recovery at day 28 (33% hypocellular, 44% normocellular, and 22% hypercellular).

**Table 3.** Clinical characteristics and bone marrow biopsy data.

White blood cell count at diagnosis (K/µL)	Median 15.3, range 0.9–360
Cytoreduction prior to induction	
Hydroxyurea	36%
Leukapheresis	3%
Days from diagnosis to treatment	Median 6, range 1–98
Inpatient days for induction	Median 27, range 6–69
% bone marrow blasts at diagnosis	Median 58%, range 11%–96%
Day 14	
% bone marrow blasts	Median 5%, range 0%–84%
Cellularity of marrow	25 Hypocellular (89%)
	One normocellular (4%)
	Two hypercellular (7%)
Day 28	
% bone marrow blasts	Median 4%, range 0%–30%
Cellularity of marrow	Nine hypocellular (33%)
	12 Normocellular (44%)
	Six hypercellular (22%)

### Adverse effects

Throughout the course of all treatments, 43% of patients required the intensive care unit (ICU), and the median days spent in the ICU, among those requiring ICU level care, was 4 days (range 1–25 days). Neutropenic fever was particularly prevalent (69%). Common infections included bacteremia (40%), pneumonia (28%), and colitis (14%). A full list of infectious events can be seen in Table 4.

**Table 4.** Adverse events throughout the course of treatment.

Required intensive care unit (ICU)	25 (43%)
Days spent in ICU	Median 4, range 1–25
Infectious events	
Neutropenic fever	40 (69%)
Bacteremia	23 (40%)
Pneumonia	16 (28%)
Colitis	8 (14%)
Abscess	6 (10%)
Cellulitis	6 (10%)
Urinary tract infection	6 (10%)
Mucositis	2 (3%)
Osteomyelitis	1 (2%)
Epiglottitis	1 (2%)
Upper respiratory infection	1 (2%)
Endocarditis	1 (2%)
Cholecystitis	1 (2%)
Appendicitis	1 (2%)
Septic arthritis	1 (2%)
Fungemia	1 (2%)
Endophthalmitis	1 (2%)

## Discussion

In this retrospective cohort of adult AML patients treated at an urban public hospital from 2014 to 2022, the CRR to induction was 51%. In other AML retrospective studies and clinical trials, CRRs to first-line therapy range from 30% to 90%^2–7,10–12,21–32^ depending on a variety of factors including intensity of regimen, age, and cytogenetic risk. Thus, the CRR observed in this cohort was comparable to previously reported rates, however lower than some seen in the highly regimented clinical trial setting where CRRs of 70%–90% have been reported.^7,10–12,25,32^ One possible explanation for the CRR of 51% observed in this retrospective cohort is that only 15% of patients who received intensive induction chemotherapy received two cycles of induction. It has been demonstrated across multiple studies that CRR is significantly better after two cycles of induction compared to one cycle.^26,30,32^

The median OS for all patients was 12.2 months with a 5-year OS of 18.2%. Surveillance, Epidemiology, and End Results (SEER) data reports a 5-year OS of 31.7%^1^ with other studies ranging from 10% to 50%.^2–6,10–12,15,16,22–29,31–38^ Survival is influenced by age, performance status, comorbidities, cytogenetic and molecular abnormalities, induction treatment, and transplant. The below-average 5-year OS observed in this retrospective cohort may have multiple contributing factors. The plurality of patients in the study had adverse risk AML (41%), and the rate of secondary AML (34%) was relatively high, both of which are associated with a poor prognosis.^23,24,28,30^ Furthermore, only 12% of patients received allogeneic SCT. Additionally, there were a substantial number of patients who received care out-of-network (47%), were lost to follow-up (14%), or received supportive care only (12%). Lastly, the patients treated at Harbor-UCLA often have complex medical comorbidities, lower socioeconomic and insurance status, difficulty navigating the healthcare system, and social barriers which limit adherence to laboratory visits and clinic appointments.

Overall, there were similarities and differences in management strategies between the patients in this study and national trends over the last decade. The majority of patients (55%) received 7 + 3 for induction, which has historically been the standard of care for fit patients. However, azacitidine + venetoclax, which is now a widely accepted regimen for older and less fit patients, was utilized relatively infrequently in the first-line setting (7%) and at relapse (12%). A possible explanation for this finding is that more than half of the study period occurred prior to the implementation of azacitidine + venetoclax as a preferred regimen for AML management.

As previously discussed, low rates of the second induction cycle and allogeneic SCT were observed. Barriers to allogeneic transplant in this population include suboptimal insurance coverage and other socioeconomic barriers, for which corrective efforts are essential and ongoing. Similar to national trends, HMA-containing regimens were widely used in the first-line (19%) and relapsed or refractory (56%) settings.

Across all patients, the most common genetic mutations were FLT3 (26%), NPM1 (24%), IDH1 (7%), TP53 (7%), IDH2 (5%), and RUNX1 (5%). The prevalence of FLT3 mutations in AML has been reported to range from 12% to 30%.^2,3,5,8,9,11,17,22,25,29,33^ FLT3 inhibitors including sorafenib, midostaurin, and gilteritinib were received by patients in this study, and newer-generation FLT3 inhibitors quizartinib and crenolanib are under investigation. The rates of IDH1 (7%) and IDH2 (5%) mutations were comparable with previously described rates ranging from 1% to 15% for each subtype.^2,3,5,7,16,17,22^ IDH1/2 inhibitors were utilized, however less frequently than FLT3 inhibitors, venetoclax, and HMAs, due to the low overall prevalence of IDH1/2 mutations. The access to targeted therapies in this retrospective cohort was encouraging, although further efforts to increase clinical trial enrollment and transplant evaluation are vital.

While this study was not aimed or powered to elucidate differences in patient traits or outcomes between ethnicity/race groups, interesting clinical information can be observed when comparing the diverse groups of patients in this cohort. Hispanic patients tended to be younger at diagnosis (median age 48 years old), a trend that has been reported previously.^17^ Among the four ethnicity/race groups, Hispanic patients had the longest median OS of 14.0 months and the highest 5-year OS at 31%. This may be related to a high rate of treatment relative to supportive care (96% vs. 4%), a robust CRR (CRR = 59%), the highest rate of allogeneic SCT (17%), and the younger age of Hispanic patients in the study.

Patients of Asian ethnicity/race had high rates of secondary AML (40%), unfavorable cytogenetics (47%), and TP53 mutations (27%). In turn, CRR (CRR = 38%) and 5-year OS (12.5%) were poor.

Black/African-American patients had a high prevalence of FLT3 mutation (33%) and the lowest rate of IDH1/2 mutations (0%). Compared to other groups, black patients had the lowest CRR (30%) and 5-year OS (0%). A significant number of black patients received only supportive care (17%), and 75% of black patients received care out-of-network throughout the course of their disease.

Patients with white/European ethnicity or race had the highest rate of secondary AML (63%) as well as the highest frequency of FLT3 mutation (38%). White/European patients had the best CRR (83%), yet this subgroup also had the highest rate of relapse (60%). Median OS for white/European patients was limited to 8.2 months with a 5-year OS of 20%.

Limitations of the present study include its retrospective nature, patient heterogeneity, and small sample size. Future efforts to incorporate AML patients from other Los Angeles County Department of Health Services hospitals may help increase the sample size and diversity of our cohort. An additional limitation of this study is the large number of patients who were lost to follow-up or received care out-of-network. This may affect both patient outcomes and the volume of analyzable patient data. Serving as a Los Angeles County safety net hospital, Harbor-UCLA sees many patients who are uninsured or underinsured, who may later proceed out-of-plan during the course of their treatment as they obtain insurance. Additionally, Harbor-UCLA provides care for many immigrant populations, some of whom may elect to return to their home country to be closer to family after diagnosis, during treatment, or while in remission.

Future aims for this retrospective cohort include increased sample size and longer follow-up. With continued advancements in AML treatments, it will be interesting to investigate their utilization in the safety net hospital setting. Further research is needed to better understand disparities in AML treatment and outcomes. Collaborative efforts to increase clinical trial enrollment and access to bone marrow transplants for minority groups and underinsured patients will be instrumental for the optimization of outcomes for all patients with AML, especially as the diversity of the United States expands and global access to cancer therapies grows.

## Abbreviations

AML: acute myeloid leukemia
OS: overall survival
HMA: hypomethylating agent
FLT3: fms related receptor tyrosine kinase 3
IDH: isocitrate dehydrogenase
MDS: myelodysplastic syndrome
MPN: myeloproliferative neoplasm
CR: complete remission
CRi: complete remission with incomplete hematologic recovery
PR: partial remission
CRR: complete remission rate
ELN: European LeukemiaNet
HIDAC: high-dose cytarabine
SCT: stem cell transplant
FLAG-IDA: fludarabine + cytarabine + granulocyte-colony stimulating factor + idarubicin
